# Ni–Al–Cr superalloy as high temperature cathode current collector for advanced thin film Li batteries†

**DOI:** 10.1039/c8ra02461h

**Published:** 2018-06-04

**Authors:** Alejandro N. Filippin, Tzu-Ying Lin, Michael Rawlence, Tanja Zünd, Kostiantyn Kravchyk, Jordi Sastre-Pellicer, Stefan G. Haass, Aneliia Wäckerlin, Maksym V. Kovalenko, Stephan Buecheler

**Affiliations:** Laboratory for Thin Films and Photovoltaics, Empa – Swiss Federal Laboratories for Materials Science and Technology Überlandstrasse 129, CH-8600 Dübendorf Switzerland nico.qca@gmail.com Stephan.Buecheler@empa.ch; Laboratory of Inorganic Chemistry, ETH Zürich Vladimir Prelog Weg 1 CH-8093 Zürich Switzerland

## Abstract

To obtain full advantage of state-of-the-art solid-state lithium-based batteries, produced by sequential deposition of high voltage cathodes and promising oxide-based electrolytes, the current collector must withstand high temperatures (>600 °C) in oxygen atmosphere. This imposes severe restrictions on the choice of materials for the first layer, usually the cathode current collector. It not only must be electrochemically stable at high voltage, but also remain conductive upon deposition and annealing of the subsequent layers without presenting a strong diffusion of its constituent elements into the cathode. A novel cathode current collector based on a Ni–Al–Cr superalloy with target composition Ni_0.72_Al_0.18_Cr_0.10_ is presented here. The suitability of this superalloy as a high voltage current collector was verified by determining its electrochemical stability at high voltage by crystallizing and cycling of LiCoO_2_ directly onto it.

## Abstract 2

A novel cathode current collector based on a Ni–Al–Cr superalloy is presented here. The suitability of this superalloy as a high voltage current collector was verified by crystallizing and cycling of LiCoO_2_ directly onto it.

## Graphical abstract

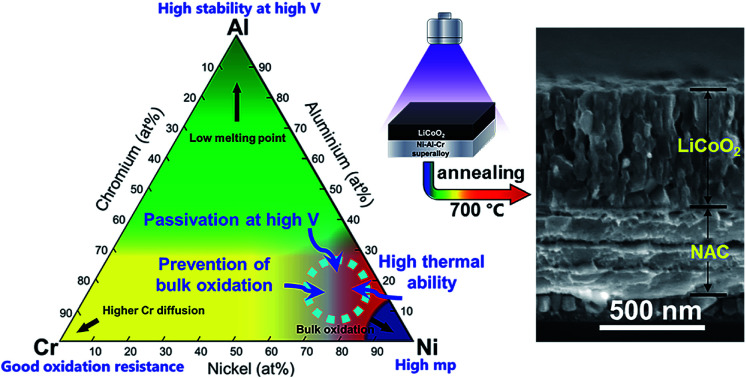

## 1. Introduction

All-solid-state lithium-based batteries (SSBs) are regarded as the next-generation energy storage solution to surpass the physicochemical limit on volumetric/gravimetric energy density and eliminate the risks from the liquid electrolyte.^1^ Bulk SSBs still face many challenges related to the electrode/electrolyte interface stability^2–5^ and engineering of the cathode,^1,6–8^ but thin film SSBs (TF-SSBs) have already demonstrated an outstanding life cycle with 90% capacity retention after 10 000 cycles using the 5 V high voltage cathode LiNi_0.5_Mn_1.5_O_4_ (LMNO).^9^ Even though the energy density of TF-SSBs is lower compared to the bulk case, they remain a promising energy storage technology for wearables, sensors, lab-on-chip, implantable and other small-sized portable devices,^10,11^ and most suitable option for applications involving flexible microdevices.^12–16^ TF-SSBs are generally produced by the sequential growth of each layer in the stack beginning by the cathode current collector (CC), comprising at intermediate stages an annealing step in air or oxygen to crystallize one or more layers.^17^ Because the optimum crystallization temperature for cathode materials such as LMNO^18^ and LiCoO_2_ (LCO),^19,20^ and fast solid-state electrolytes such as the garnet-type Li_7_La_3_Zr_2_O_12_ (LLZO) and perovskite-type Li_0.17_La_0.61_TiO_3_ (LLTO) are at least 700–750 °C in order to get the desired phases and attain high ionic conductivity.^21–24^ The commonly used CCs such as Al cannot withstand such high temperatures in an oxidizing atmosphere without melting or becoming highly insulating. Stainless steels (SSs), on the other hand, are a tempting choice due to their oxidation resistance at high temperature, but it has been shown for LiMn_2_O_4_ that both Fe and Cr diffuse into the cathode degrading its performance.^25^ Similar shortcomings are observed for LCO on SS and are reported in this work.

In a previous work we introduced Cr_2_N as a high-temperature cathode CC. Even though the material showed feasibility up to 700 °C, we could observe a substantial Cr diffusion at 600 °C.^26^ To improve the performance at high temperature, Ni-based superalloys are promising candidates for oxidation resistant and high melting point CCs. In the case of Ni–Al–Cr superalloys as the one developed in the present work, benefits arise from combining Al, with well-known self-passivating properties as well as high stability at high voltage once passivated,^27^ and Cr with good oxidation resistance at high temperature. However, the incorporation ratios should be carefully considered to avoid thick insulating layer formation or out-discusion issues for following cathode material. It has been reported that more than 16 wt% (30 at%) of Al in Ni–Al alloys leads to a parabolic scale growth of a continuous insulating Al_2_O_3_ passivation layer.^28^ As demonstrated by Han *et al.* the incorporation of Al into LCO begins already at 400 °C, although after long annealing periods of 8 hours, while in Mn-based cathode materials Al does not diffuse in but results in the formation of the electrical insulator LiAlO_2_ at 600 °C.^29^ On the other hand, Cr has a detrimental effect on the performance of LiCoO_2_ when it forms LiCo_1−*x*_Cr*_x_*O_2_ with *x* = 0.2 or above,^30^ hindering the use of this element alone with Ni. Thus, we selected the Al and Cr-deficient composition Ni_0.72_Al_0.18_Cr_0.10_ as target composition. The simultaneous incorporation of Cr and Al in a Ni superalloy has a synergetic effect on the oxidation resistance, decreasing the minimum amount of these alloying elements required to prevent bulk oxidation compared to Ni–Cr and Ni–Al binary alloys.^31^ The tunable composition can enlarge the electrochemical window together with a reduced diffusion of Cr and Al into the cathode at 700 °C. We demonstrate a thermally stable Ni–Al–Cr current collector on which LCO can readily crystallize, achieving 80% of the theoretical capacity even without major optimization of the LCO deposition conditions.

## 2. Experimental

The Ni–Al–Cr (NAC) alloy was deposited as alternating layers of Al, Ni and Cr by electron beam evaporation in a BAK UNI evaporator (evatec) at a pressure of 2 × 10^−4^ Pa, using a deposition rate of 5 Å s^−1^ for Al, 10 Å s^−1^ for Ni and 1 Å s^−1^ for Cr. The Ni–Al–Cr multilayer was later annealed in vacuum (<5 × 10^−2^ Pa) at 700 °C for 6 hours in a tube furnace (Carbolite GHA 12/300) to alloy the multilayer. For characterization purposes, MgO-coated silicon wafers (100, boron doped silicon, Prime grade, University wafers) are used as substrate. The MgO interlayer, which prevents the reaction between NAC and silicon at high temperature, was sputtered from a MgO target (99.99% purity, Stanford advanced materials) at a pressure of 0.6 Pa using an Ar to O_2_ ratio of 60 : 0.5, power density of 3.12 W cm^−2^, substrate temperature of 100 °C and substrate-to-target distance of 10 cm. MgO was later annealed to 850 °C for 3 hours in air using a heating/cooling ramp of 5 °C per minute to crystallize it.

LCO was deposited by RF magnetron sputtering in an Orion sputtering system (AJA International) in confocal off-axis geometry from a LiCoO_2_ target (99.9% purity, Edgetech Industries LLC). Three different deposition conditions were employed in order to improve the LCO performance denoted as LP LCO-Ar, LP LCO-O2 and HP LCO-O2. The detailed fabrication parameters are listed in Table 1. All LCO thin films were annealed in air at 700 °C for 1 hour with a heating/cooling ramp of 5 K min^−1^ in a chamber furnace (Heraeus K114). The total quantity of cathode material in each cell was determined by weighing each substrate before and after deposition of LCO with an analytical balance (Mettler Toledo XS205 Dual Range, 0.01 mg readability).

**Table 1:** The deposition parameters of the LiCoO_2_ thin films

Condition	Pressure (Pa)	Working gas	Ratio (sccm)	Add substrate bias of 50 V	Target-to-substrate distance (cm)	Deposition rate (Å min^−1^)
LP LCO-Ar	4 × 10^−2^	Ar	1	No	11.8	11
LP LCO-O2	0.79	Ar : O_2_	24 : 1	Yes	11.8	4
HP LCO-O2	3	Ar : O_2_	24 : 1	Yes	9.8	<2.5

SE (secondary electron) and BSE (back-scattered electron) images were acquired in a scanning electron microscope (Hitachi FEG-SEM S-4800) working at 5 kV. Energy dispersive X-ray spectroscopy (EDX) spectra were registered at the indicated accelerating voltages using a Bruker XFlash 6|10 X-ray detector with 121 eV resolution at Mn Kα and the standardless quantification was performed with the software ESPRIT from Bruker. X-ray diffractograms were obtained with a Bruker D8 Discover diffractometer using Cu Kα1 radiation in a grazing incidence configuration.

Raman measurements were performed between 300 and 800 cm^−1^ with a 50× objective, using a Renishaw Raman system equipped with a HeNe Laser, *λ* = 633 nm.

X-ray photoelectron spectroscopy (XPS) measurements were performed with monochromatized Al Kα X-ray source (PHI Quantum 2000 equipment) at room temperature. The surveys were acquired with 117.4 eV pass energy and single scans with 29.35 eV pass energy. The later provides high-resolution scans with the full-width half maximum of 1 eV. Calibration was performed with the C 1s peak at 285.0 eV. Depth profiles were carried out with an Ar^+^ ion sputter gun, operated with an acceleration voltage of 2 kV.

For the determination of the resistivity, gold contacts of ∼70 nm thickness, spaced as in Fig. S3,† were deposited by thermal evaporation on NAC (420 nm) on sapphire ((0001) orientation, Stettler sapphire). The gold (4N purity) was evaporated at a pressure of 2 × 10^−4^ Pa and a rate of 1.3 Å s^−1^ as determined by a quartz crystal microbalance, without intentionally heating the substrates. The area comprising the gold contacts was scribed with a diamond tip in order to minimize edge effects. The resistance between each pair of contacts was determined from the *I*–*V* curves using a Keithley 2400 source meter by four-point probe measurements at room temperature and in air. The sample resistance was obtained by the transmission line method. The resistivity was then calculated by multiplying by the contact length (8 mm) and layer thickness (420 nm).

Impedance measurements were conducted using a PAIOS 4.0 system (Fluxim AG). Samples were measured in an LTSE-420-P temperature-controlled stage (Linkam Scientific Instruments Ltd) and the temperature monitored using a PT-100 sensor attached to the sample. The impedance response was measured in the range 10 mHz to 10 MHz with an oscillation amplitude of 10 mV in the temperature range from 297.8 K to 346.9 K in dry air atmosphere, while the fitted range was 10 mHz to 1 MHz. All impedance spectra analysis and equivalent circuit simulation were carried out in ZView 2.0 (Scribner Associates, Inc.). Impedance data were validated using a linear Kramers–Kronig method.^32^

Elemental characterizations were performed at the 3 MV tandem accelerator of the National Center for Accelerators (Sevilla, Spain) by proton elastic backscattering spectroscopy (p-EBS). p-EBS was performed with a proton beam of 3.0 MeV and a passivated implanted planar silicon (PIPS) detector at a 160° scattering angle. The Rutherford Backscattering (RBS) spectra were analyzed with the SIMNRA software^33^ after subtracting the substrate contribution. Non-Rutherford cross sections were used in the software for Li and O elements.

Secondary ion mass spectrometry (SIMS) measurements were recorded on a TOF-SIMS system from ION-TOF using O_2_^+^ primary ions with 2 keV of ion energy, a current of 400 nA, and a raster size of 400 × 400 μm^2^. An area of 100 × 100 μm^2^ was analyzed using Bi^+^ ions with 25 keV of ion energy.

Electrochemical measurements were conducted in air tight coin-type cells, assembled in an Ar-filled glove box (O_2_ < 0.1 ppm, H_2_O < 0.1 ppm). Elemental lithium was employed as both reference and counter electrode in Li half cells. As electrolyte 1 M LiPF_6_ (battery grade, Novolyte) in dimethyl carbonate and ethylene carbonate (1 : 1 EC/DMC by weight, Novolyte) with 3 wt% 4-fluoro-1,3-dioxolan-2-one (FEC, battery grade, Solvionic) was used. Pre-dried glass microfiber (GF/D, Whatman, 80 °C for 12 hours under vacuum) served as separator. LCO cells were cycled between 3–4.25 V *vs.* Li/Li^+^. All electrochemical measurements were carried out at room temperature on MPG2 multi-channel workstation (BioLogic). Specific capacities and currents were quantified with respect to the mass of the electrode loading.

The electrochemical stability of current collectors was probed by cyclic voltammetry measurements in 1 M LiPF_6_ in EC/DMC electrolyte at scan rate of 0.1 mV s^−1^. The measurements were conducted in Ar-filled glove box (O_2_ < 0.1 ppm, H_2_O < 0.1 ppm) using elemental lithium and tungsten as reference and counter electrodes, respectively.

## 3. Results and discussion

**Fig. 1:**
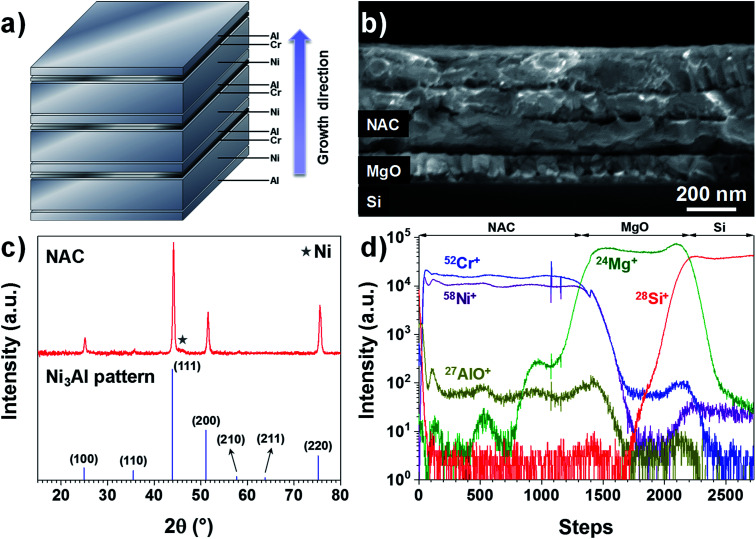
(a) Scheme of the multilayer growth sequence and the thickness of each metal layer. (b) SEM image of the NAC multilayer deposited on MgO/Si (100) after annealing at 700 °C for 6 hours in vacuum. (c) GIXRD diagram of the annealed Ni–Al–Cr multilayer deposited on sapphire glass. (d) TOF-SIMS depth profile of NAC (420 nm)/MgO/Si for ^58^Ni^+^, ^52^Cr^+^, ^27^Al^16^O^+^, ^24^Mg^+^ and ^28^Si^+^.

The as-deposited Ni–Al–Cr multilayer consists of 10 sequentially evaporated layers as schematized in Fig. 1a in order to facilitate the intermixing of the metals upon annealing. As observed in Fig. 1b, after annealing for 6 hours at 700 °C only 3 fused layers are visible, which is consistent with a Ni-rich alloy. If a fused single layer is intended, then higher temperatures are needed or other deposition techniques such as sputtering should be used. Here we restricted the annealing temperature to 700 °C in order to reduce alloying between the NAC and the SS substrate in later experiments.

**Fig. 2:**
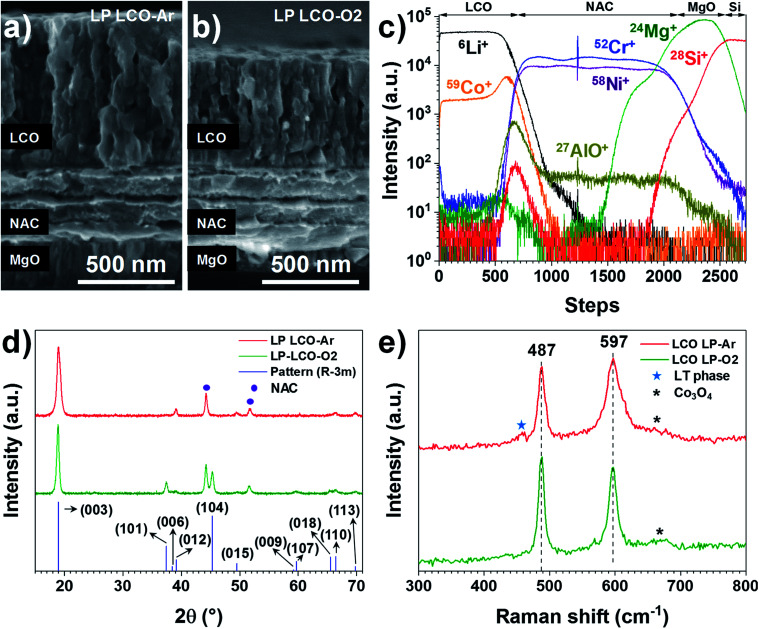
SEM cross-section of (a) LP LCO-Ar and (b) LP LCO-O2 after annealing at 700 °C in air for 1 hour. (c) TOF-SIMS depth profile of LP LCO-Ar (∼200 nm)/NAC (420 nm)/MgO/Si for ^6^Li^+^, ^59^Co^+^, ^58^Ni^+^, ^52^Cr^+^, ^27^Al^16^O^+^, ^24^Mg^+^, ^28^Si^+^ and ^7^Li^27^Al^16^O^+^. (d) GIXRD of annealed LP LCO-Ar and LP LCO-O2 on NAC (420 nm)/MgO/Si, and the reference LiCoO_2_ pattern (ICSD database, collection code 48 103). (e) Raman spectrum of annealed LP LCO-Ar and LP-LCO-O2 on NAC (420 nm)/MgO/Si.

Taking into account the nature of the SS used in this work (Cr content between 16–18.5%), we have prepared an alloy with the target composition Ni_0.72_Al_0.18_Cr_0.10_, which corresponds to weight percentage of 81% of Ni, 9% of Al and 10% of Cr, using a multilayer approach as depicted in Fig. 1a (SEM image presented in Fig. S1†). For the sake of simplicity, this alloy will be called NAC in the following. Once annealed the NAC layer still presents a multilayered structure (Fig. 1b) mainly due to the high melting point of Ni (1453 °C). The estimated composition from EDX measurements (Table ST1†) is Ni_0.73±0.06_Al_0.20±0.01_Cr_0.07±0.01_. The calculated density for such composition is approximately 8.4 g cm^−3^, which is considerably lighter than Pt (21.45 g cm^−3^) normally used as cathode current collector in Li-based thin film batteries.^19,34^

The electrical resistivity for a 420 nm NAC film is (1.25 ± 0.06) × 10^−6^ Ω m as determined by four-point probe measurements using the transmission line method (Fig. S3†). This value is higher than for NiCr alloys (∼1.1–1.2 × 10^−6^ Ω m when Cr is up to 30 wt%^35^) and even higher than for Ni_3_Al alloys (∼4.4 × 10^−7^ Ω m (ref. 36)), which can be attributed to the simultaneous incorporation of Cr and Al into the superalloy. Moreover, the XRD diffractogram in Fig. 1c indicates that the multilayer forms predominantly the solid solution Ni_3_Al (ICSD 260169) with a small contribution of Ni (Fig. S4†) as indicated by the star, which is in perfect agreement with the work performed by Merchant *et al.*^37^ on the phase diagrams of the Ni–Al–Cr system, indicating an effective alloying of the multilayer. The difference in peaks intensity and slight shift with respect to the Ni_3_Al reference pattern can be attributed to the excess of Ni and incorporation of Cr. The TOF-SIMS depth profile in Fig. 1d shows no strong gradient in the profiles for Ni^+^, Cr^+^, and AlO^+^ (Al signal was saturating). Despite the visible oscillations of the signal, no significant variations in concentration of Al and Cr along the NAC are expected due to the absence of their respective diffraction peaks in the XRD of Fig. 1c.

Both LP LCO-Ar (Fig. 2a) and LP LCO-O2 (Fig. 2b) grown onto the NAC by RF magnetron sputtering show high porosity upon one hour annealing at 700 °C in air and a columnar structure which is more evident for higher thicknesses (Fig. S5†). The TOF-SIMS depth profile for LP LCO-Ar in Fig. 2c does not show severe diffusion of Li and Co into NAC after the annealing process at 700 °C in air for 1 hour. Due to the porous nature of the annealed LCO (Fig. 2a), a broadening of the attributed interfacial region by TOF-SIMS can be expected. The LP LCO-Ar was chosen on purpose for this study due to the high lithium content of the as-prepared film with a Li/Co ratio of ∼1.4, as calculated from RBS experiments (Fig. S6a and Table ST2†). Moreover, both Cr^+^ and AlO^+^ signals only show low intensity in the LCO region indicating limited diffusion into the cathode material.

The annealing of LP LCO-Ar films leads to crystalline LCO with preferred orientation in the (003) direction (Fig. 2d), and no secondary phases such as Co_3_O_4_ are observable. On the other hand, LP LCO-O2 exhibits a less intense (003) diffraction peak and a noticeable (104) reflection (Fig. 2d). For the HP LCO-O2 the (003) reflection in the XRD of Fig. S7b† is completely absent, but the intense peaks at 31.3° and 36.8° are a clearer indication of unwanted formation of secondary phase Co_3_O_4_. In addition, the SEM cross-section of HP LCO-O2 in Fig. S7a† points out a phase segregation which correlates well with the formation of Co_3_O_4_. It is generally hypothesized that the (003) texture, contrary to the (101) and (104) ones, does not provide facile diffusion paths for Li ions,^38–41^ so a negative impact on cathode performance can in principle be expected even for the LP LCO-O2. While in HP LCO-O2 the (003) diffraction peak is absent, the presence of the secondary phase Co_3_O_4_ might have a detrimental effect on the ionic conductivity of the cathode.

The main peaks in the Raman spectrum of LP LCO-Ar/NAC/MgO/Si in Fig. 2e (top) correspond to the high-temperature phase of LCO,^19^ but in this case some broad bands around 620–700 cm^−1^ are an indication of Co_3_O_4_,^42^ which is expected to form due to the low sputtering pressure employed, while the band located at 440–470 cm^−1^ and the lifted background between the two main peaks are mainly attributed to the low-temperature LCO phase.^42^ As demonstrated by H. Y. Park *et al.*, S. Tintignac *et al.* and C.-L. Liao *et al.*, the effect of pressure,^40,42^ bias^43^ and temperature^19^ have a dramatic impact on the properties of LCO and its capacity, where sputtering pressures lower than 3 Pa (4 × 10^−2^ Pa used for LP LCO-Ar) and the absence of bias lead to overstoichiometric LCO films (Li rich)^40,43^ and Co_3_O_4_ formation^42,43^ which has negative effect on the capacity of the cathode.^42,43^ For LP LCO-O2 the two main peaks in the Raman spectrum (Fig. 2e, bottom) become thinner, while the peak at 597 cm^−1^ is now less intense compared to the peak at 487 cm^−1^, an indication of a lower amount of the low-temperature phase.^42^ The bump at ∼670 cm^−1^ has been assigned by Tintignac *et al.* to Co_3_O_4_,^42^ and can be attributed to an unbalanced Li/Co ratio,^40,42^ which for annealed LP LCO-O2 films is ∼1.1 (Fig. S6b and Table ST2†).

**Fig. 3:**
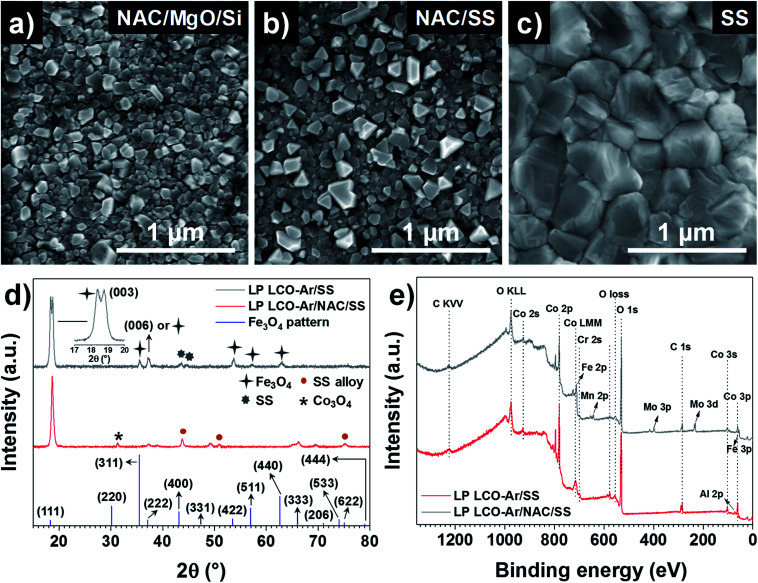
SEM top view of LP LCO-Ar annealed at 700 °C in air for 1 hour on (a) NAC/MgO/Si, (b) NAC/SS and (c) SS. (d) GIXRD at *ω* = 2° of annealed LP LCO-Ar on SS and NAC/SS, and the reference Fe_3_O_4_ pattern (ICSD database, collection code 35 001). (e) XPS survey of annealed LP LCO-Ar (∼200 nm) on SS and NAC/SS.

SEM top view images in Fig. 3a–c show the surface morphology of LP LCO-Ar annealed at 700 °C in air for 1 hour on different substrates. If grown on NAC/MgO/Si (Fig. 3a) small irregular crystals ≤100 nm dominate the surface of LCO, while in the case of NAC/SS (Fig. 3b) there are also larger dispersed crystals segregated on the surface which can be attributed to Cr diffusion as reported in our previous work.^26^ This is further supported by the XPS depth profile for Cr in Fig. S8.† For NAC/MgO/Si Cr signal is in the noise level throughout the LCO layer (Fig. S8a†), whereas for NAC/SS the Cr signal is more intense close to the surface and decreases towards the current collector (Fig. S8b†). On the other hand, on SS (Fig. 3c) considerably larger grains (>300 nm) are mostly observed.

According to the XRD figures of annealed LP LCO-Ar on NAC/SS (Fig. 3d), the NAC diffraction peaks are shifted to lower angles (43.74°) due to the alloying with SS (named as SS alloy in the figure). Moreover, we attribute the small peak at 31.28° to Co_3_O_4_ (Fig. 3d). The amount of Fe incorporated into NAC estimated by EDX is around 14–15%, while there is also an increase of the at% of Cr (Fig. S2 and Table ST1†). Despite the meaningful amount of incorporated Fe (but considerably lower than for SS), the surface chemistry of these alloys is still determined by the selective oxidation of Al and, to a lower extent, Cr.^44,45^ On SS, however, besides the main (003) reflection, numerous peaks which we attribute to Fe_3_O_4_ can be observed (Fig. 3d). The XPS surveys in Fig. 3e indicate the presence of Cr in LCO (∼200 nm) annealed on NAC/SS, while a small signal for Al and none for Fe can be identified (Fig. 3e). Furthermore, the position of the Al 2p peak at 73.0 eV is too low in energy to correspond to Al_2_O_3_ (74.3 eV) or LiAlO_2_ (74.1 eV) and it has been assigned in literature to LiCo_1−*x*_Al_*x*_O_2_.^46,47^ On the other hand, the surface survey for LCO (∼200 nm thick) annealed on SS indicates diffusion of Fe, Cr, Mn and Mo, which can lead to the formation of large crystals on the surface by heterogeneous nucleation (Fig. 3c).^26^ In both surveys contribution from the substrate cannot be completely discarded due to the porosity of LCO and eventual cracks. However, it can be expected that the majority of the signal arises from the LCO layer.

**Fig. 4:**
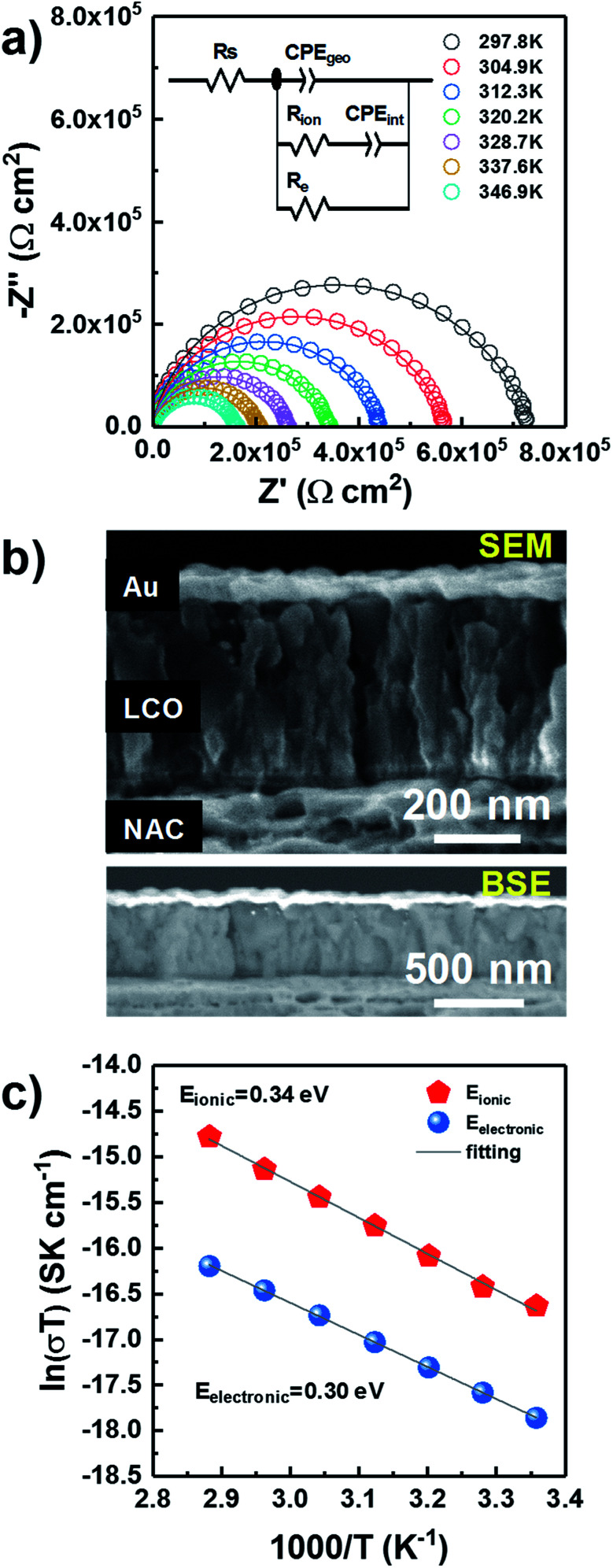
Through-plane impedance measurements of LP LCO-O2 (∼430 nm)/NAC (420 nm)/sapphire after annealing at 700 °C in air for 1 hour. (a) 297.8–346.9 K Nyquist plots including the equivalent circuit used for the fitting (solid line), R: resistor, CPE: constant phase element, (b) SEM (top) and BSE (bottom) cross section of Au/LP LCO-O2/NAC/sapphire, and (c) Arrhenius-type plot in the same temperature range.

Through-plane impedance measurements were carried on gold-coated LP LCO-O2 (∼430 nm)/NAC/sapphire as shown in Fig. 4. This LCO was chosen due to its better electrochemical performance (see discussion below). A simple Debye circuit does not yield a satisfactory fitting of the Nyquist plot. As reported in literature, a resistor needs to be added in parallel to account for the mixed conductor behavior (*i.e.* a combination of ionic and electronic conductivities) of LCO.^48^ This results in the depicted equivalent circuit (Fig. 4a), composed of a geometric capacitance in parallel with a series *R*_ion_–*C*_int_ configuration (modeling ionic conductivity and interfacial capacitance through the material) and a resistor *R*_e_ (modeling electronic conductivity).^48^ In the fitting, constant phase elements (CPE) are used instead of ideal capacitors to model the non-idealities of the interfacial charge transfer (see ESI† for more details on the interpretation). A series resistance *R*_s_ is added to account for the contact resistance.

The effects of ionic transport through grain boundaries are not observed in the impedance spectrum, which can be explained from the columnar morphology of LCO (Fig. 2a and S5†) and hence its low number of grain boundaries in the through-plane direction. In addition, no meaningful variation in the impedance is expected from the evaporated gold on the sample due to its negligible penetration into LCO as observed in the SEM (Fig. 4b top) and BSE (Fig. 4b bottom) images.

The evolution of the impedance as temperature increases is plotted in Fig. 4a, where a noticeable shrinking of the arcs is observed for higher temperatures. After verifying that the data satisfied the Kramers–Kronig conditions, with deviations below 2% over the whole frequency range (Fig. S9†), all data were fitted with the equivalent circuit in Fig. 4a. The values of the different fitted parameters are reported in Table ST3† along with a brief discussion concerning their physical meaning. The electronic and ionic conductivity values for the measured temperature range are summarized in Table ST4,† yielding ∼0.6 × 10^−10^ S cm^−1^ and ∼2.0 × 10^−10^ S cm^−1^ at RT, respectively. Although these seem rather low values, it is difficult to establish any comparison with other works due to the lack of literature related to direct measurements of LCO thin films, without any delithiation treatment by liquid electrolyte or conductive additive. But it is worth mentioning that the fully lithiated LiCoO_2_ is at least 4 orders of magnitude less electronically conductive in the majority of published literature.^49–51^ Moreover, the preferred orientation and film porosity may also influence the measured conductivity.^52,53^ According to our previous results, our sputtered LCO thin presents (003) preferred orientation and highly porous morphology. All of these factors may play a role in the low electronic conductivity of the measured LCO.

Two activation energies can be differentiated in the Arrhenius-type plot (Fig. 4c) with values of 0.34 eV and 0.30 eV for the ionic and electronic transport, respectively (Table ST5†). These values are in agreement with previously reported ones in literature (0.2–0.45 eV) associated to the electronic and ionic transport phenomena in LCO.^49,54–59^ Notice that this value is considerably lower than for LiAlO_2_, which lays above 0.7 eV in the temperature range RT-200 °C,^60^ discarding the formation of this compound in a significant amount in agreement with Han *et al.*^29^ and XPS (Fig. 3e). Moreover, the estimated series resistance at room temperature is ∼2.3 Ω cm^2^ (Table ST3†), suggesting a good ohmic contact between LCO and NAC, while the absence of a high capacitance in the impedance spectra (Fig. 4a) also discards the formation of a thick insulating Al_2_O_3_ layer at the NAC-LCO interface.

**Fig. 5:**
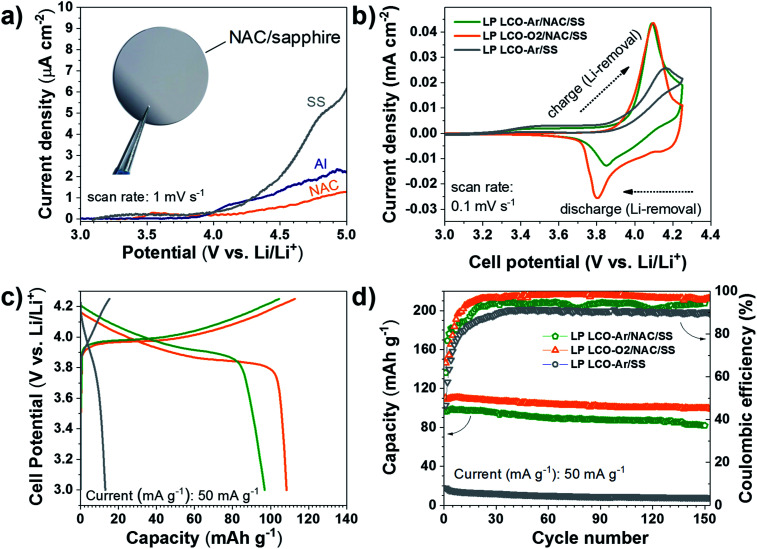
(a) Voltamperometry of SS, Al and NAC, inset: photograph of NAC on sapphire, (b) cyclic voltammograms of LCO on SS and NAC/SS measured between 3–4.25 V *vs.* Li/Li^+^. (c) Galvanostatic charge and discharge curves of LCO on SS and NAC/SS. (d) Cycling behavior of LCO measured at the same C rate on SS and NAC/SS.

The electrochemical response of the NAC layer was determined in LiPF_6_ electrolyte in order to assess its stability at high voltage. As seen in Fig. 5a the current density observed with NAC is significantly lower than that of SS and lower also than of Al, which is used as standard current collector in most Li-based batteries. Although Cr and Ni are not stable up to 5 V *vs.* Li/Li^+^ in standard liquid-based electrolyte,^61^ the passivated Al with alumina or AlF_3_ formed by reaction with the electrolyte serves as good passivation layers at high voltage.^61^ This explains the observed superior stability of the NAC layers since aluminum is dominating the surface chemistry when oxidation processes are involved.

The electrochemical behavior of LCO not only depends on the sputtering conditions^42,43,62^ but also on the substrate used as support as observed in the cyclic voltammograms of Fig. 5b. LP LCO-Ar on bare SS presents a quite poor electrochemical response in the potential range 3–4.25 V *vs.* Li/Li^+^. On the other hand, LP LCO-Ar on NAC exhibits better-defined peaks. Furthermore, for the more optimized deposition conditions of LP LCO-O2 (Fig. 5b), attained by increasing the pressure, introducing a small amount of oxygen and adding a bias, the shape of the peaks become more pronounced. Our results are in line with literature where it was demonstrated that an increase in pressure and the use of a bias led to better-defined peaks and an overall improved electrochemical response.^19,40,42,43^

The galvanostatic charge and discharge curves of Fig. 5c evidence an extremely low capacity for LCO on SS of ∼13 mA h g^−1^ even for a ∼200 nm thick layer. Kalaiselvi *et al.* observed a steadily decrease in the capacity of LiCo_1−*y*_Fe_*y*_O_2_ upon incorporation of higher amounts of iron (up to *y* = 0.4), accompanied by lower coulombic efficiencies, although no explanation for this behavior was given.^63^ However, here we attribute such low capacity to the formation of Fe_3_O_4_ (Fig. 3c–e) rather than substitution of Co by Fe in LCO, which exhibits electrochemical response below 2.5 V *vs.* Li/Li^+^. The capacity of LCO on NAC is significantly increased from ∼97 mA h g^−1^ (LP LCO-Ar) to ∼108 mA h g^−1^ (LP LCO-O2), Fig. 5c. A capacity fade is observed in all cases (Fig. 5d) which is usual in these thin film systems coupled to a liquid electrolyte.^19,42,43^ The HP LCO-O2 sputtered using optimum deposition conditions (according to literature) yielded a much lower capacity of ∼72 mA h g^−1^ (Fig. S10†) which is in agreement with the more intense Co_3_O_4_ peak in XRD (Fig. S7b†). This difference with regard to literature is attributed to our confocal off-axis sputtering configuration, limiting the deposition rate in HP LCO-O2 below 2.5 Å min^−1^. Such low rates can lead to substantial re-sputtering during the deposition process yielding more Co_3_O_4_ as observed here. Moreover, the O_2_ partial pressure employed in this work was below 1 mbar, however a higher pressure would be desirable to avoid O_2_ deficiency in the sputtered films, but this would lead in our system to even lower rates and higher re-sputtering.^19^

## 4. Conclusions

In this study, we report a high temperature stable Ni–Al–Cr superalloy with resistivity 1.25 × 10^−6^ ± 6 × 10^−8^ Ω m for target composition Ni_0.72_Al_0.18_Cr_0.10_. The detailed TOF-SIMS and XPS indicate that the Ni–Al–Cr superalloy exhibited restricted Al and Cr elements out-diffusion into the LiCoO_2_ cathode and suppressed Fe and Cr diffusion from the stainless steel substrate when NAC was implemented as a diffusion barrier on SS after 700 °C annealing.

Impedance spectroscopy analysis at different temperatures for LCO on NAC show the activation energies of 0.30 eV (electronic) and 0.34 eV (ionic), excluding LiAlO_2_ formation as a result of the high temperature processing, while the low series resistance of 2.3 Ω cm^2^ also excludes the presence of a thick insulating Al_2_O_3_ layer at the LCO/NAC interface.

The suitability as high voltage current collector is verified by the low current density (<2 μA cm^−2^) observed at 5 V *vs.* Li/Li^+^ in the first scan. The electrochemical performance of LCO processed on NAC/SS is greatly superior to that on bare SS, achieving an initial discharge capacity of ∼108 mA h g^−1^ and ∼13 mA h g^−1^, respectively. In order to further improve the performance of LCO on NAC it is mandatory to optimize the deposition conditions of the former, eliminating the (003) texture while minimizing Co_3_O_4_ formation.

## Conflicts of interest

There are no conflicts to declare.

## Supplementary Material

RA-008-C8RA02461H-s001
