# Supplementary material for: Ni–Al–Cr superalloy as high temperature cathode current collector for advanced thin film Li batteries
Source: RSC Adv. 2018 Jun 4;8(36):20304–13. doi: 10.1039/c8ra02461h (PMC9080811; doi:10.1039/c8ra02461h)
Supplement: RA-008-C8RA02461H-s001 [file RA-008-C8RA02461H-s001.pdf]

Electronic Supplementary Material (ESI) for RSC Advances.
This journal is © The Royal Society of Chemistry 2018

Electronic Supplementary Information

Ni-Al-Cr superalloy as high temperature cathode current collector for advanced thin film Li batteries

Alejandro N. Filippin,[#a] Tzu-Ying Lin,[#a] Michael Rawlence,[a] Tanja Zünd,[a,b] Kostiantyn Kravchyk,[a,b] Jordi Sastre-Pellicer,[a] Stefan G. Haass,[a] Aneliia Wäckerlin,[a] Maksym V. Kovalenko,[a,b] Stephan Buecheler*[a]

[a] Laboratory for Thin Films and Photovoltaics, Empa - Swiss Federal Laboratories for Materials Science and Technology, Überlandstrasse 129, CH-8600 Dübendorf, Switzerland.

[b] Laboratory of Inorganic Chemistry, ETH Zürich, Vladimir Prelog Weg 1, CH-8093 Zürich, Switzerland

*contact e-mail: nico.qca@gmail.com, Stephan.Buecheler@empa.ch

[#] equal contribution to this work

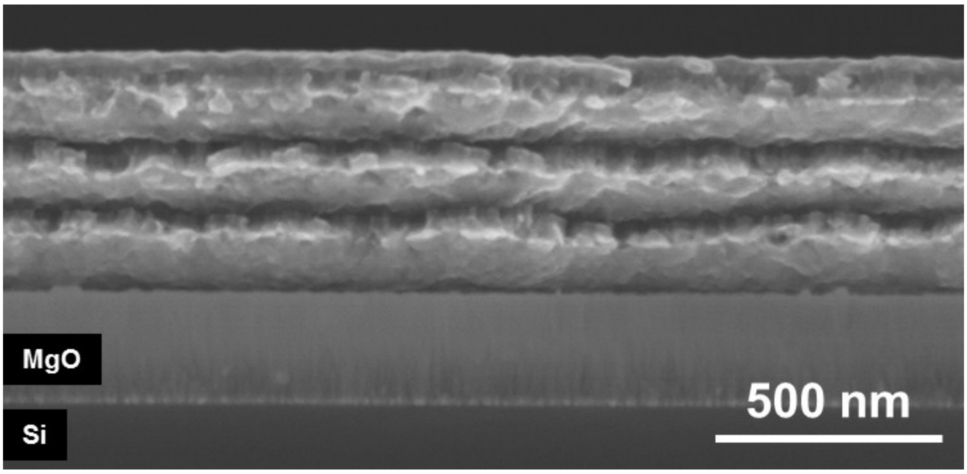

Figure S1. SEM cross-section of the as-prepared Ni-AlCr multilayer prepared by e-beam evaporation.

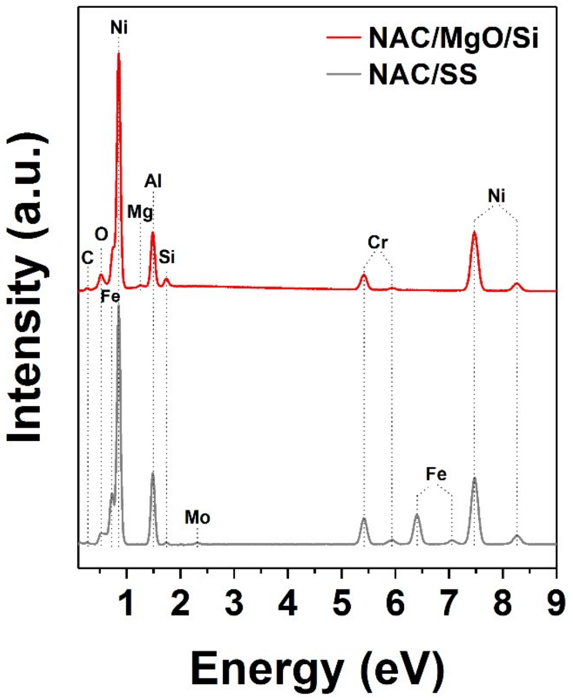

Figure S2. Normalized EDX spectra obtained at 15 kV of of NAC(420 nm) on MgO/Si (red) and on SS (grey), after annealing in vacuum at 700 °C for 6 hours,

Table ST1. Quantification values of NAC(420 nm) on MgO/Si and on SS, after annealing in vacuum at 700 °C for 6 hours, from the EDX spectra acquired at 15 kV accelerating voltages (crack free zone).

	Element	At. No.	Netto	Mass [%]	Mass Norm. [%]	Atom [%]	abs. error [%]	rel. error [%]
NAC/MgO/Si	Carbon	6	6784	2.50	2.21	9.49	0.34	13.43
	Oxygen	8	15201	1.67	1.67	4.75	0.22	13.12
	Nickel	28	344566	77.34	77.29	60.04	2.38	3.07
	Aluminum	13	166902	9.61	9.61	16.23	0.47	4.88
	Chromium	24	76575	6.97	6.97	6.11	0.22	3.22
	Magnesium	12	8838	0.69	0.69	1.29	0.06	8.90
	Silicon	14	25933	1.28	1.28	2.08	0.08	6.09
			Sum	100.06	100	100		
NAC/SS	Carbon	6	2141	0.77	0.77	3.23	0.14	17.57
	Oxygen	8	6179	0.69	0.69	2.18	0.11	16.16
	Nickel	28	200496	64.80	64.78	55.62	2.00	3.08
	Aluminum	13	111429	8.15	8.15	15.22	0.40	4.93
	Chromium	24	71737	9.32	9.31	9.03	0.29	3.13
	Silicon	14	2006	0.13	0.13	0.23	0.03	23.52
	Molybdenum	42	2312	0.22	0.22	0.11	0.03	14.96
	Iron	26	83863	15.96	15.95	14.40	0.49	3.07
			Sum	100.04	100	100		

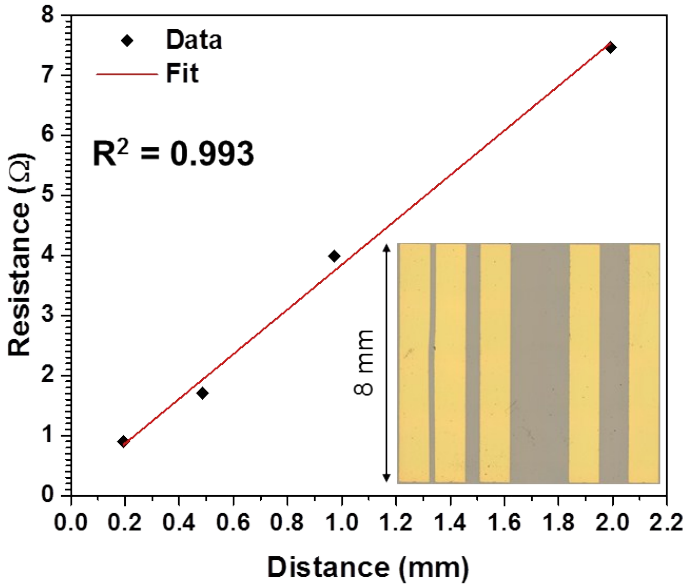

Figure S3. Resistance versus distance for 420 nm NAC obtained by four-point probe measurements at room temperature. Inset: Optical microscope image of the 150 nm thick gold contacts on NAC/sapphire.

The reference pattern presented in Figure S4 for Ni (collection code 58038) was taken from the ICSD database.

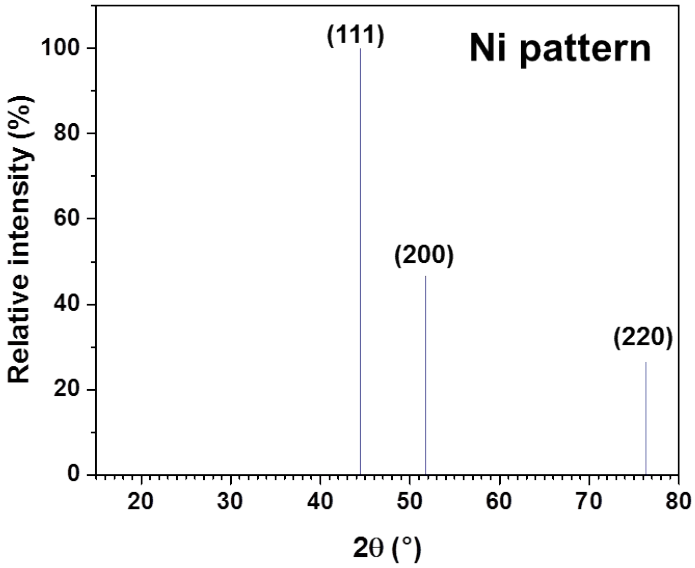

Figure S4. Ni reference diffraction pattern.

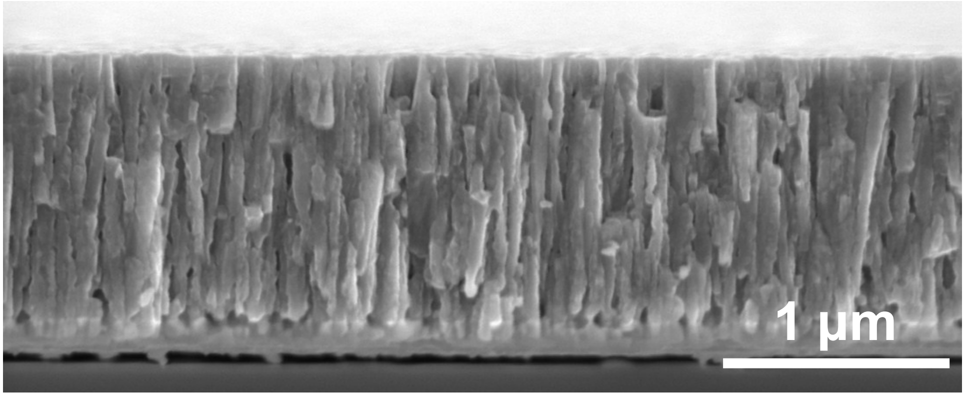

Figure S5. SEM cross-section of ~ 1.2 µm LP LCO-O2 annealed on MgO/Si annealed at 700 °C in air for 1 hour.

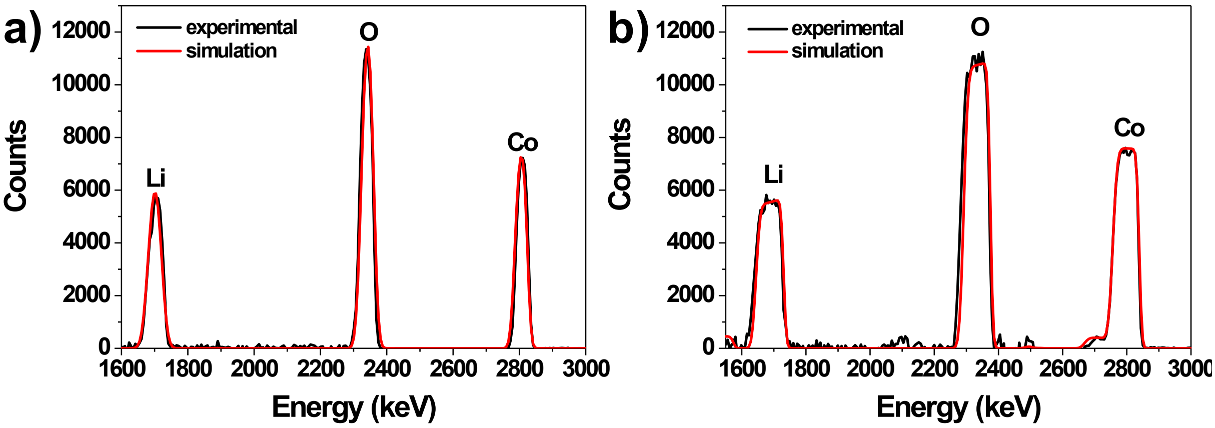

Figure S6. RBS spectra of a) as-deposited LP LCO-Ar and b) LP LCO-O2 annealed at 700 °C in air for 1 hour (both on MgO) after subtraction of the background.

Table ST2. Quantification calculated from the spectrum in Fig. S6 using the SIMNRA software.

Sample	Li (10^{15}at/cm^2)	Co (10^{15}at/cm^2)	O (10^{15}at/cm^2)	Li (at. %)	Co (at. %)	O (at. %)
As-prepared LP LCO-Ar	1810 ± 90	1285 ± 65	3170 ± 158	28.9 ± 1.4	20.5 ± 1.0	50.6 ± 2.5
Annealed LP LCO-O2	3190 ± 159	6135 ± 306	2925 ± 146	26.0±1.3	50.1 ± 2.5	23.9 ± 1.2

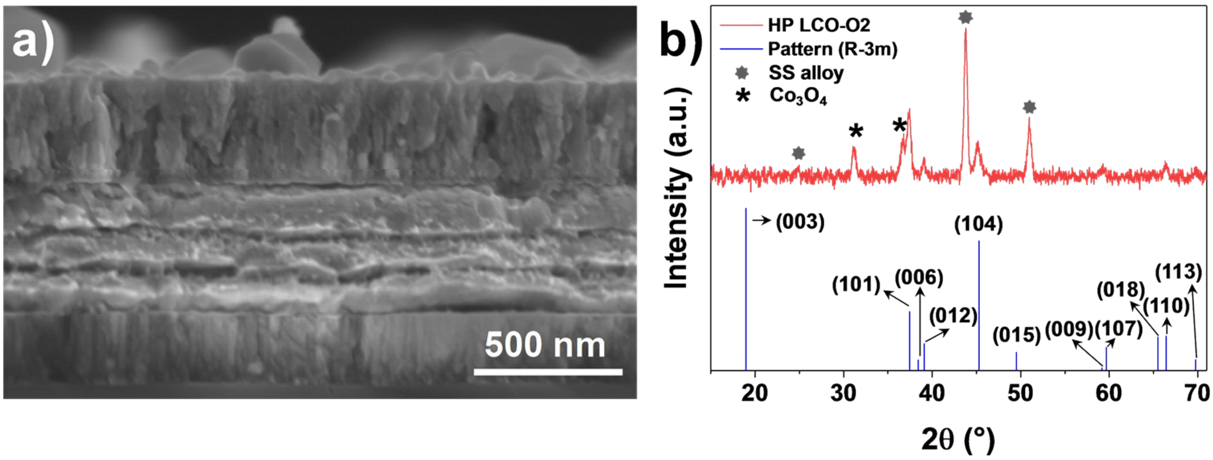

Figure S7. SEM cross-section of HP LCO-O2/NAC/MgO/Si after annealing at 700 °C in air for 1 hour (a) and its corresponding GIXRD compared to the $LiCoO_2$ pattern (ICSD database, collection code 48103) (b).

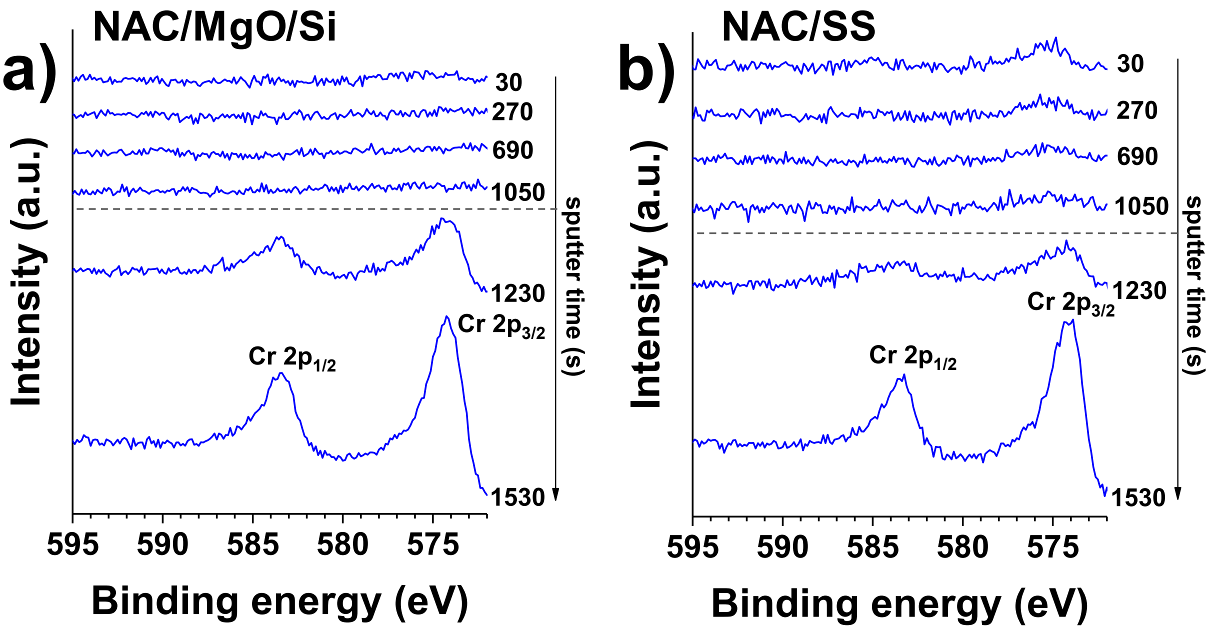

Figure S8. XPS depth profile for Cr of LP LCO-Ar (~ 200 nm) after annealing at 700 °C in air for 1 hour on NAC/MgO/Si (a) and NAC/SS (b). The dashed line represents the interface between the current collector and LCO.

Table ST3 contains the parameters that result from fitting the equivalent circuit in Figure 4a to the measured impedance spectroscopy data, and the fitting errors (chi-squared and sum of squares). A decrease of the ionic and electronic resistances is observed as the temperature becomes higher, which corresponds to the increase of both ionic and electronic conductivities. The magnitude (Q) of the geometric constant phase element (CPE_{geo}) remains mostly constant over the whole temperature range, only increasing slightly due to the change in the geometry of the sample upon thermal expansion. The exponent factor (n) of CPE_{geo} is close to 1 in all fits, indicating that it is close to an ideal capacitor. In exchange, the CPE modeling the interface capacitance (CPE_{int}) has an exponent factor around 0.65, because of

the non-ideal interface between LCO and the electrodes. The magnitude of CPE_{int} varies over temperature, due to the temperature dependence of the electrochemical processes that take place at the interfaces. Finally, a fluctuation of the series resistance (Rs) is also observed. This may be due to a change in the contact between the contacting needles and the electrodes as a result of the temperature change.

Table ST3. Fitting values for the impedance of Au/LP LCO-O2 (435 nm)/NAC/sapphire at different temperatures in Figure 4c using the equivalent circuit from Figure 4a.

Temp. (K)	Chi-Sqr	Sum-Sqr	R_s (Ω)	R_{ion} (Ω)	CPE_{geo}-Q (F)	CPE_{geo}-n	R_e (Ω)	CPE_{int}-Q (F)	CPE_{int}-n
297.8	2.49E-04	3.36E-02	14.32	1.36E+06	2.54E-08	9.66E-01	4.62E+06	2.97E-08	6.51E-01
304.9	2.57E-04	3.47E-02	14.25	1.12E+06	2.59E-08	9.65E-01	3.59E+06	3.26E-08	6.62E-01
312.3	2.73E-04	3.68E-02	18.21	8.25E+05	2.64E-08	9.65E-01	2.79E+06	3.69E-08	6.61E-01
320.2	2.25E-04	3.04E-02	36.17	6.07E+05	2.71E-08	9.65E-01	2.17E+06	4.32E-08	6.51E-01
328.7	2.47E-04	3.34E-02	44.4	4.55E+05	2.78E-08	9.64E-01	1.66E+06	4.72E-08	6.58E-01
337.6	2.69E-04	3.64E-02	92.14	3.44E+05	2.89E-08	9.62E-01	1.29E+06	5.43E-08	6.53E-01
346.9	2.90E-04	3.91E-02	57.53	2.48E+05	2.96E-08	9.62E-01	1.02E+06	6.20E-08	6.52E-01

Table ST4. Calculated conductivity values from the resistances in Table ST3, electrode geometry 0.8x0.2 cm^2 and LCO layer thickness of 435 nm.

Temp. (K)	297.8	304.9	312.3	320.2	328.7	337.6	346.9
σ_{ion} (S cm^{-1})	2.00E-10	2.42E-10	3.30E-10	4.48E-10	5.98E-10	7.89E-10	1.10E-09
σ_e (S cm^{-1})	5.9E-11	7.6E-11	9.8E-11	1.26E-10	1.64E-10	2.10E-10	2.67E-10

Table ST5. Fitting values for the activation energy of Au/LCO/NAC/sapphire (Fig. 4d). k_B is Boltzmann constant.

	E_{ionic}	$E_{electronic}$
Equation	y = a + b*x	
x	1000/T	
y	ln(σT)	
a	-3.43 ± 0.26	-6.06 ± 0.06
b	-3.95 ± 0.08	-3.51 ± 0.02
R-Square	99.78%	99.99%
Ea = -1000*k_B*b (eV)	**0.34±0.01**	**0.30±0.00**

The observed feature in all K-K residuals around 10 kHz is due to a change in the transimpedance amplifier gain of the measurement system.

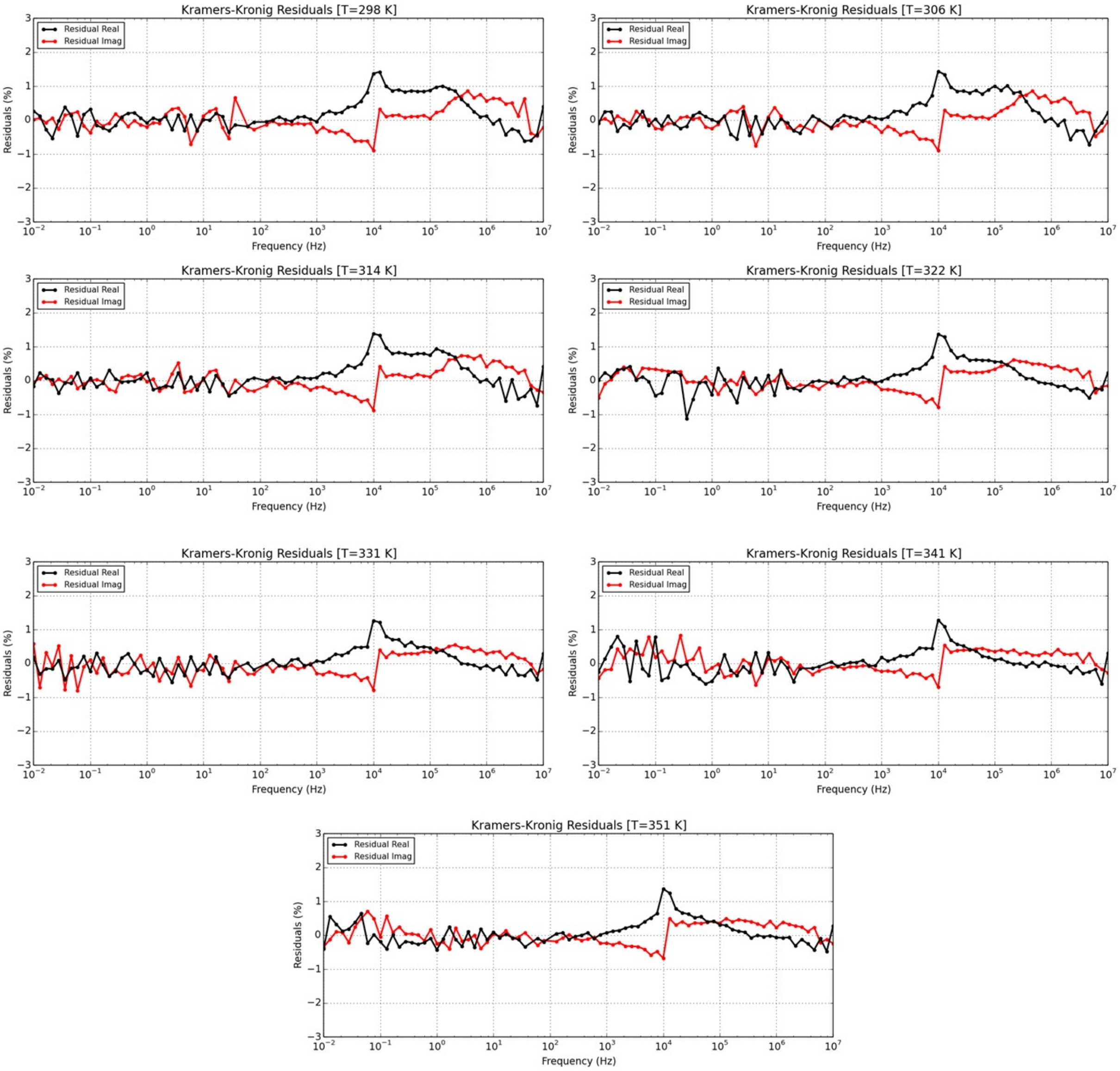

Figure S9. Kramers-Kronig test for all temperatures measured of the LP LCO-O2 (~ 430 nm) / NAC (420 nm) / sapphire.

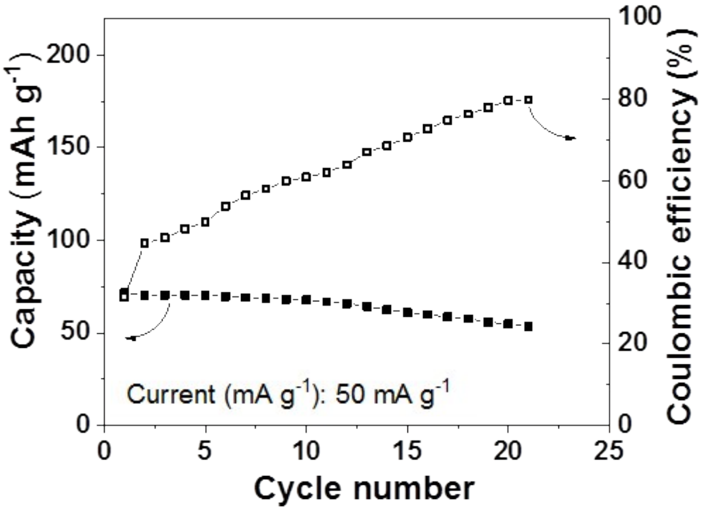

Figure S10. Cycling behavior of HP LCO-O2 measured at 50 mA g^{-1} rate on and NAC/SS.
